# Early Elevation of Systemic Plasma Clusterin after Reperfused Acute Myocardial Infarction in a Preclinical Porcine Model of Ischemic Heart Disease

**DOI:** 10.3390/ijms21134591

**Published:** 2020-06-28

**Authors:** Denise Traxler, Andreas Spannbauer, Patrick Einzinger, Julia Mester-Tonczar, Dominika Lukovic, Johannes Winkler, Katrin Zlabinger, Alfred Gugerell, Ljubica Mandic, Mariann Gyöngyösi, Noemi Pavo

**Affiliations:** 1 Division of Cardiology, Department of Internal Medicine II, Medical University of Vienna, 1090 Vienna, Austria; denise.traxler-weidenauer@meduniwien.ac.at (D.T.); andreas.spannbauer@meduniwien.ac.at (A.S.); julia.mester-tonczar@meduniwien.ac.at (J.M.-T.); dominika.lukovic@meduniwien.ac.at (D.L.); johannes.winkler@univie.ac.at (J.W.); katrin.zlabinger@meduniwien.ac.at (K.Z.); alfred.gugerell@meduniwien.ac.at (A.G.); ljubica.mandic@meduniwien.ac.at (L.M.); noemi.pavo@meduniwien.ac.at (N.P.); 2 Institute of Information Systems Engineering, Research Unit of Information and Software Engineering, Vienna University of Technology, 1040 Vienna, Austria; patrick.einzinger@tuwien.ac.at

**Keywords:** acute myocardial infarction, clusterin, NGS, miRNAs

## Abstract

Clusterin exerts anti-inflammatory, cytoprotective and anti-apoptotic effects. Both an increase and decrease of clusterin in acute myocardial infarction (AMI) has been reported. We aimed to clarify the role of clusterin as a systemic biomarker in AMI. AMI was induced by percutaneous left anterior artery (LAD) occlusion for 90 min followed by reperfusion in 24 pigs. Contrast ventriculography was performed after reperfusion to assess left ventricular ejection fraction (LVEF), left ventricular end diastolic volume (LVEDV) and left ventricular end systolic volume (LVESV) and additional cMRI + late enhancement to measure infarct size and LV functions at day 3 and week 6 post-MI. Blood samples were collected at prespecified timepoints. Plasma clusterin and other biomarkers (cTnT, NT-proBNP, neprilysin, NGAL, ET-1, osteopontin, miR21, miR29) were measured by ELISA and qPCR. Gene expression profiles of infarcted and remote region 3 h (*n* = 5) and 3 days (*n* = 5) after AMI onset were analysed by RNA-sequencing. AMI led to an increase in LVEDV and LVESV during 6-week, with concomitant elevation of NT-proBNP 3-weeks after AMI. Plasma clusterin levels were increased immediately after AMI and returned to normal levels until 3-weeks. Plasma NGAL, ET-1 and miR29 was significantly elevated at 3 weeks follow-up, miR21 increased after reperfusion and at 3 weeks post-AMI, while circulating neprilysin levels did not change. Elevated plasma clusterin levels 120 min after AMI onset suggest that clusterin might be an additional early biomarker of myocardial ischemia.

## 1. Introduction

Early diagnosis and therapy of acute myocardial infarction (AMI) are essential to reduce infarct size and improve prognosis [1,2,3]. Current gold standard of early diagnosis of AMI is high-sensitivity cardiac troponin (hs-cTn), [4] even though this marker has several limitations [5]. It has restricted specificity with an increase in non-ischemic injuries, such as pulmonary oedema, or embolisation, anticancer treatment or chronic kidney disease [6]. Therefore, further research to identify novel biomarkers that may be used alone or in combination with already established markers are necessary to improve diagnosis of AMI allowing rapid adequate therapy.

Clusterin (also known as apolipoprotein J) is a heat shock protein-like extracellular protein that is expressed in a variety of human tissues and body fluids [7]. Clusterin is involved in cell differentiation, cell-cycle regulation, DNA repair, transcription, apoptosis, lipid transportation, tissue remodelling and cell-cell or cell-substrate interactions [8,9]. Its physiological role depends on isoforms, localisation and cell type origin; however, it can exert anti-inflammatory, cytoprotective and anti-apoptotic effects [10,11,12]. Clusterin levels are up-regulated under conditions of cell stress and tissue injury and in patients suffering from myocardial infarction, dialyses-related amyloidosis, atherosclerosis, cancer, diabetes and neurodegenerative diseases [9]. In the early and later stage of myocardial ischemia, plasma clusterin levels are found to be both increased and decreased [8,13]. Furthermore, elevated plasma clusterin levels are associated with left ventricular (LV) remodelling [14]. Additionally, clusterin was expressed at a higher percentage in the infarct and border zone tissue [15,16]. Besides a diagnostic role, clusterin has also been applied therapeutically in animal studies [17].

Up to now, several plasma biomarkers were investigated to be associated with AMI, such as (neprilysin, neutrophil gelatinase-associated lipocalin [NGAL], osteopontin or endothelin-1 [ET-1]). Neprilysin is a zinc-dependent endopeptidase that has been found in a variety of tissues, e.g., brain, lungs, heart, kidney, testes, adipose tissue, endothelial cells, vascular smooth muscle cells and cardiac myocytes [18,19]. It is involved in insulin regulation, inflammation control, natriuretic peptide degradation in cardiovascular disease and beta-amyloid degradation in the brain [20]. Recent studies indicate a relationship between neprilysin and adverse outcome in acute and chronic heart failure [21,22]. However, in AMI patients, no dynamics of neprilysin was observed, nor did plasma neprilysin correlate with infarct size or ejection fraction [18]. NGAL is a 25 kDa large glycoprotein that is released by a plethora of cell types (among others cardiomyocytes) [23,24]. High levels are reported in coronary artery disease and are associated with disease instability [25]. Moreover, increased NGAL levels are associated with poor mortality in acute coronary syndrome [26]. NGAL was also elevated with developing chronic myocardial hypertrophy in an experimental model of artificial aortic isthmus stenosis [27]. Osteopontin is a matrix cellular protein released from cells during tissue injury and remodelling [28]. Under healthy conditions, the heart does not express osteopontin; however, in AMI tissue, resident macrophages express osteopontin [29,30]. Suezawa et al. reported that plasma osteopontin levels were elevated after AMI and remained high until day 14 [31]. ET-1 is one of the most potent vasoconstrictors known that is particularly expressed by endothelial cells [32]. Plasma ET-1 levels are increased in cardiovascular disease, e.g., unstable angina pectoris, AMI and heart failure and are associated with clinical outcome [33,34].

Myocardial ischemia is linked to LV remodelling, a process that is characterised by cardiomyocytes hypertrophy, cardiac fibrosis and deformation of the heart chambers [35]. Cardiac fibrosis is an important pathophysiological transformation, as abundant fibrosis may result in ventricular dilatation, enlarged infarct zones and chronic heart failure [36]. miR-21 and miR-29 are supposed to exert profibrotic effects [37,38]. miR-21 has been intensively investigated in cardiovascular diseases. Elevated miR-21 expression is not only associated with worse cardiac function, but has also been targeted as a therapeutic agent. However, recent data on miR-21 show contradictory results, so that both antagomiRs and Ad-miR-21 have been used to treat LV remodelling [39,40,41]. Down-regulation of miR-29 has been clearly associated with fibrotic states not only in the heart [38]. In the healthy heart miR-29 levels are high in order to balance ECM turnover [42].

Based on contradictory data on the above-mentioned biomarkers in AMI we aimed to further clarify the dynamics of clusterin, neprilysin, NGAL, osteopontin, ET-1, miR21 and miR29 in a preclinical porcine model of reperfused AMI in which duration and extent of myocardial ischemia are standardised and comparable, and associated with cardiac magnetic resonance imaging (cMRI)-derived cardiac function parameter.

## 2. Results

### 2.1. Myocardial Necrosis and Functional Parameters by Cardiac MRI

**Figure 1:**
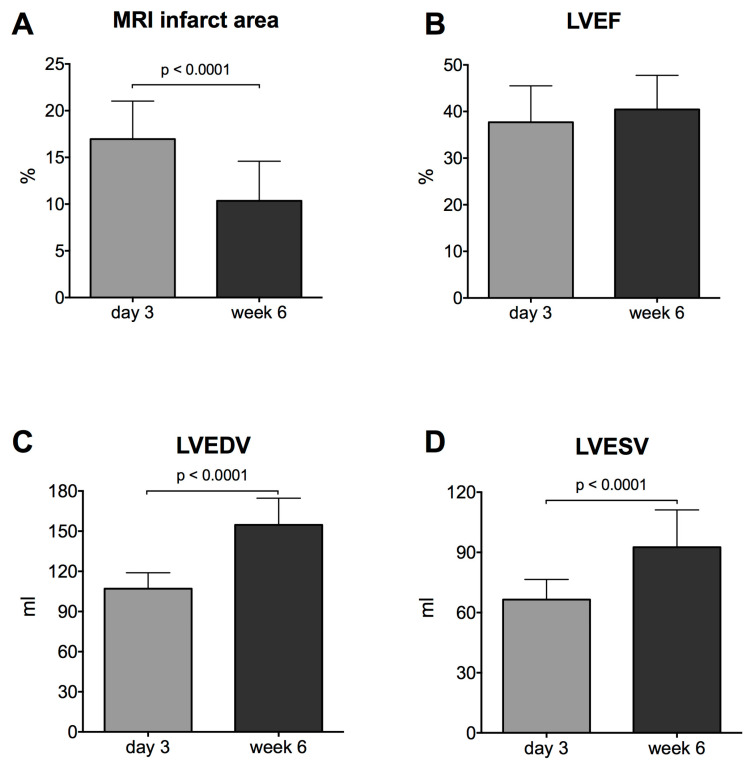
Infarct size (**A**), left ventricular ejection fraction (LVEF) (**B**), left ventricular end diastolic volume (LVEDV) (**C**) and left ventricular end systolic volume (LVESV) (**D**) 3 days and 6 weeks after acute myocardial infraction (*n* = 24).

To quantify myocardial necrosis as well as left ventricular dysfunction, we assessed infarct size, left ventricular ejection fraction (LVEF), left ventricular end systolic volume (LVESV) and left ventricular end diastolic volume (LVEDV) using cMRI at 3 days as well as 6 weeks after reperfused AMI. Myocardial necrosis (as % of LV) was decreased at 6 weeks as compared to 3 days, most probably due to shrinkage of the scar tissue (mean ± SD: 17.0 ± 4.1 vs. 10.4 vs. 4.2%, *p* < 0.0001, Figure 1A). LVEDV and LVESV increased at week 6 (mean ± SD: LVEDV: 107.0 ± 11.9 vs. 154.7 ± 19.9 mL, *p* < 0.0001, Figure 1C; LVESV: 66.5 ± 10.06 vs. 92.6 ± 18.6 mL, *p* < 0.0001, Figure 1D), in accordance to the natural growth of the domestic pigs. The AMI-induced decrease in LVEF was not changed between 3 days and 6 weeks post-AMI (mean ± SD: 37.7 ± 7.82 vs. 40.5 ± 7.3%, *p* = 0.15, Figure 1B).

### 2.2. Plasma Clusterin after Acute Myocardial Infarction

**Figure 2:**
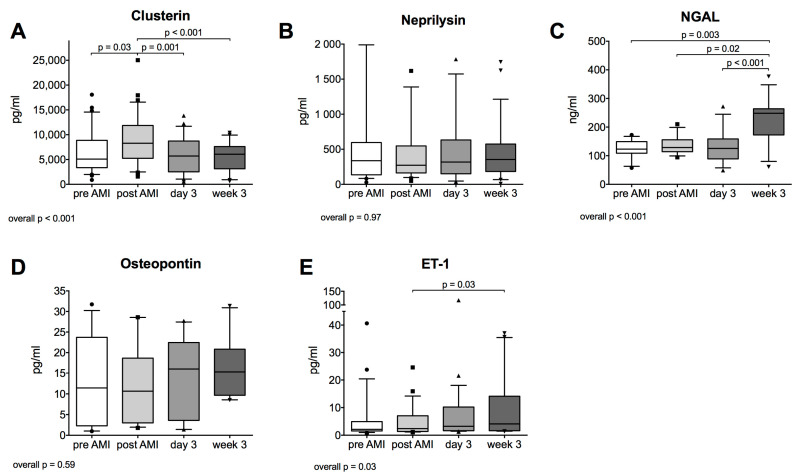
Plasma Clusterin (**A**), Neprilysin (**B**), neutrophil gelatinase-associated lipocalin (NGAL) (**C**), Osteopontin (**D**) and endothelin-1 (ET-1) (**E**) before acute myocardial infarction (AMI), after AMI, on day 3 and week 3 after AMI (*n* = 24).

Plasma clusterin levels were increased 120 min after infarction onset (*p* = 0.03) and significantly decreased afterwards at day 3 (*p* = 0.001) and week 3 (*p* < 0.001) (compared to post AMI value) (median [IQR]: pre vs. post vs. 3 d vs. 3 w: 4952.0 [3389.0; 8413.1] vs. 7982.4 [5587.0; 11,840.5] vs. 5701.4 [2521.2; 8670.5] vs. 6069.6 [3182.2; 7491.5] pg/mL, *p* < 0.001, Figure 2A).

### 2.3. Other Biomarkers Associated with Myocardial Ischemic Injury

Plasma neprilysin levels did not change after AMI (median [IQR]: pre vs. post vs. 3 d vs. 3 w: 336.6 [136.1; 595.6] vs. 272.8 [162.2; 548.0] vs. 318.2 [157.5; 630.9] vs. 354.9 [185.2; 573.5] pg/mL, *p* = 0.97, Figure 2B). Plasma NGAL concentration significantly increased at 3 weeks after AMI (median [IQR]: pre vs. post vs. 3 d vs. 3 w: 122.9 [109.1; 145.5] vs. 128.5 [114.9; 155.0] vs. 125.4 [89.9; 151.2] vs. 248.4 [178.4; 262.5] pg/mL, *p* < 0.001, Figure 2C). Osteopontin plasma levels remained equal after myocardial infarction compared to baseline levels during the whole follow up period (median [IQR]: pre vs. post vs. 3 d vs. 3 w: 11.4 [2.3; 23.7] vs. 10.7 [3.0; 18.7] vs. 16.0 [3.6; 22.5] vs. 15.3 [9.7; 20.8] pg/mL, *p* = 0.59, Figure 2D). For ET-1, we could observe increased concentration 3 weeks after myocardial infarction (median [IQR]: pre vs. post vs. 3 d vs. 3 w: 2.1 [1.5; 4.9] vs. 2.4 [1.4; 7.0] vs. 3.2 [1.6; 10.2] vs. 4.1 [1.6; 14.3] pg/mL, *p* = 0.03, Figure 2E).

**Figure 3:**
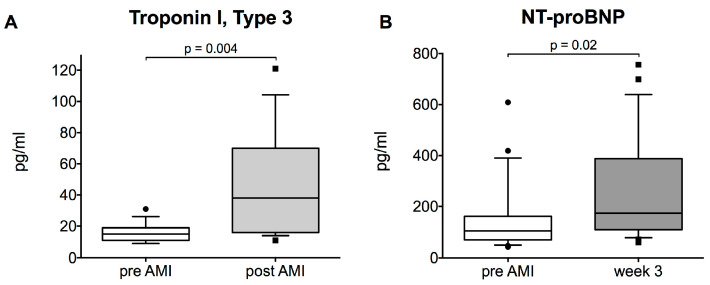
Troponin I, Type 3 pre-acute myocardial infarction (AMI) and 120 min after AMI onset (post-AMI) (**A**) and NT-proBNP before and 3 weeks after AMI (**B**) (*n* = 24).

Troponins are currently gold standard in diagnosis of AMI. We measured Troponin I, type 3 and observed an increase 120 min after AMI onset (median [IQR]: pre vs. post: 15.0 [11.0; 19.0] vs. 38.0 [16.0; 70.0] pg/mL, *p* = 0.004, Figure 3A).

NT-proBNP is an established marker of myocardial dysfunction and prognosis in chronic heart failure [43]. We assessed plasma NT-proBNP levels, additionally to cMRI to evaluate chronic LV dysfunction after AMI. We observed a significant increase after 3 weeks (median [IQR]: pre vs. 3 w: 112.3 [71.3; 167.9] vs. 189.9 [109.9; 387.9] pg/mL, *p* = 0.02, Figure 3B).

### 2.4. Pro-Fibrotic Plasma miR21 and miR29 after Acute Myocardial Infarction

**Figure 4:**
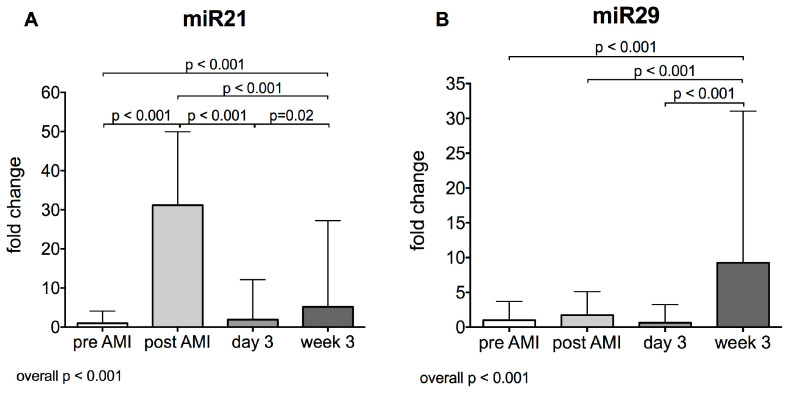
miR21 (**A**) and miR29 (**B**) before acute myocardial infarction (AMI), after AMI, on day 3 and week 3 after AMI (*n* = 24).

LV remodelling and cardiac fibrosis are highly relevant pathological processes after AMI and for a variety of miRNAs, an association with cardiac fibrosis has been observed. Relative expression of both miR21 and miR29 increased at 3 weeks compared to baseline levels and relative expression of miR21 was also significantly increased post reperfusion compared to baseline (mean ± SD: pre vs. post vs. 3 d vs. 3 w: miR21: 1.0 ± 3.1 vs. 31.2 ± 18.8 vs. 1.9 ± 10.2 vs. 5.2 ± 22.0 fold increase, *p* < 0.001, Figure 4A; miR29: 1.0 ± 2.7 vs. 1.7 ± 3.4 vs. 0.6 ± 2.6 vs. 9.3 ± 21.8 fold increase, *p* < 0.001, Figure 4B).

### 2.5. Association between Clusterin and Left Ventricular Function Parameters, Infarct Size and Biomarkers

**Figure 5:**
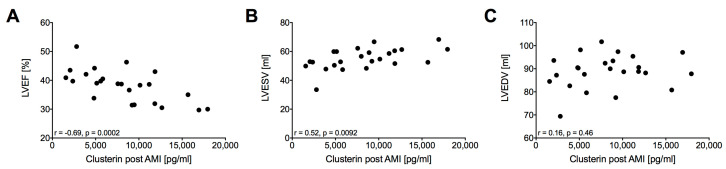
Correlation of plasma clusterin post AMI with LVEF (**A**), LVESV (**B**) and LVEDV (**C**) 120 min after AMI onset (*n* = 24).

We correlated clusterin 120 min after AMI onset with LV function parameters assessed by contrast ventriculography. Plasma clusterin post AMI was significantly associated with LVEF (r = −0.69, *p* = 0.0002, Figure 5A) and LVESV (r = 0.52, *p* = 0.0092, Figure 5B), but not LVEDV (r = 0.16, *p* = 0.46, Figure 5C). Significant negative correlation was also found between the changes of clusterin from pre-AMI to post-AMI with 3 d cMRI LVEF (r = −0.544, *p* = 0.036). Clusterin levels at day 3 and week 3 follow-up did not correlate with the 3 day and 6 week left ventricular function parameters and infarct size.

Additionally, elevated, clusterin concentration at 120 min post AMI-onset did not correlate with plasma levels of any other assessed biomarker, including Troponin I, type 3 (r = −0.21, *p* = 0.33) and miR21 (r = −0.31, *p* = 0.14).

### 2.6. Transcriptomic Profiling

**Figure 6:**
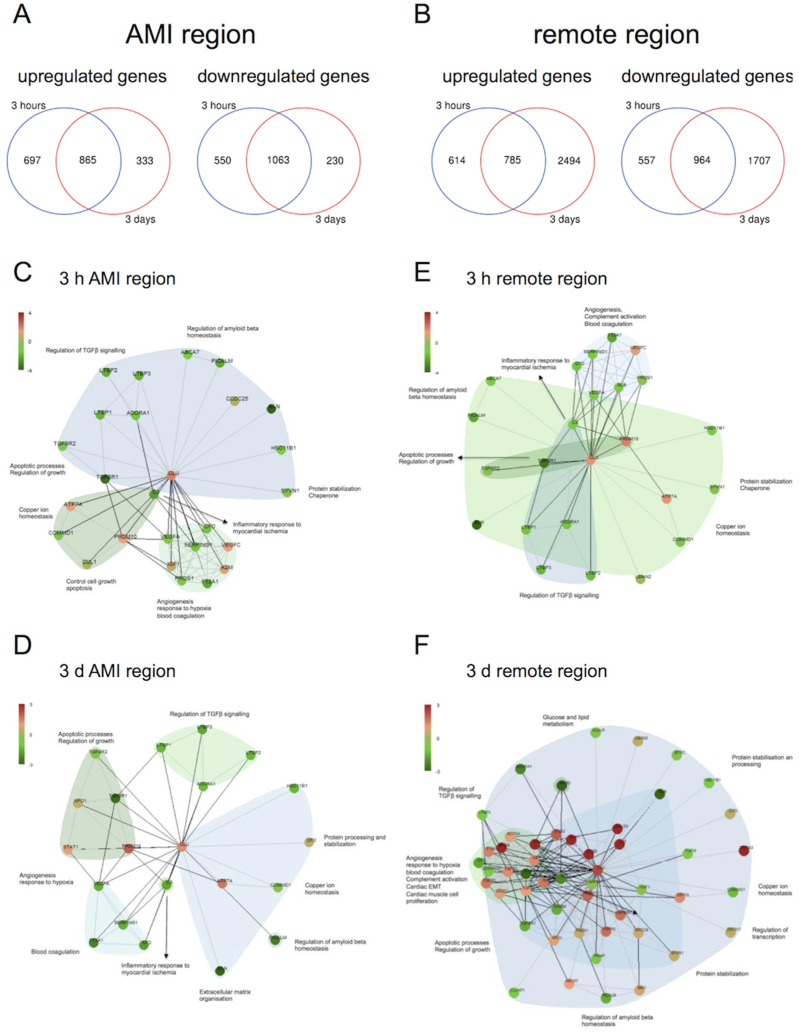
Venn diagrams with the number of significantly up- and downregulated protein coding genes in the groups 3 h and 3 days after myocardial infarction onset, related to controls in the AMI (**A**) and remote region (**B**). Protein-protein interactions of the differentially expressed genes focused on CLU in AMI tissue at 3 h (**C**) and 3 days (**D**) and remote tissue 3 h (**E**) and 3 days (**F**) after AMI onset with their main functional classes. CLU was upregulated both 3 h and 3 days after myocardial infarction onset in AMI tissue and remote region. Red, upregulated; green, downregulated genes, *n* = 15.

Venn diagrams (Figure 6A,B) reveal a notable overlap, but also differences between up- and down regulated genes 3 h and 3 days after AMI onset both in AMI and remote region. Clusterin was significantly upregulated in AMI and remote tissue both after 3 h and 3 days. Functional clustering focussed on clusterin as a central gene (Figure 6C,D) showed a strong connection to genes associated with angiogenesis, complement activation (inflammatory response to myocardial ischemia), apoptotic processes, TGFβ signalling, amyloid beta homeostasis and other genes involved in protein stabilization and chaperoning. The majority of those genes are downregulated. A similar pattern was observed 3 h after AMI onset in the remote region (Figure 6E). A cluster of mostly upregulated genes associated with angiogenesis, complement activation and cardiac muscle cell proliferation was identified in the remote region on day 3 (Figure 6F). A direct comparison of genes involved in cardiac muscle function and apoptosis in AMI and remote region both after 3 h and 3 days is given in the Supplementary Material online (Table S1).

## 3. Discussion

In the present study, we demonstrate increased plasma clusterin levels after ischemic myocardial injury (120 min after AMI onset) that returned to normal levels already after 3 days, suggesting that it can be a similar and additionally early and high-sensitive biomarker of acute myocardial ischemia as hs-cTn, which increases 2–3 h after ischemia onset and remains elevated several days after AMI. Furthermore, we observed a correlation of plasma clusterin 120 min after AMI onset with LVEF and LVESV. The failure of correlation between clusterin (a molecular chaperone) and TnI levels might be explained by the different intracellular localization, subcellular compartments and molecular interactions, and warrants further investigations.

In the current clinical setting, biomarkers play an important role in diagnosis and early treatment modalities of AMI. Even though hs-cTn has improved diagnosis and management of AMI patients, they still feature several drawbacks such as limited specificity and a delay in measurably increased values [6]. Diagnosis of AMI with hs-cTn might be difficult in patients suffering from chronic renal failure, subarachnoid haemorrhage, acute pulmonary embolism, chronic obstructive pulmonary disease, acute noncardiac critical illness and after strenuous exercise. Furthermore, an elevation of hs-cTn by other cardiac causes such as advanced heart failure, direct myocardial trauma, acute pericarditis, acute inflammatory myocarditis and tachycardia has been observed [44]. This clearly indicates that there is a dearth of biomarkers that could identify myocardial ischemia within the first few hours after the onset of AMI additionally to hs-cTn. In our study, acute myocardial ischemia did not alter the plasma neprilysin and osteopontin concentration, while NGAL, ET-1 and miR29 increased at week 3 post-AMI, suggesting a role in development of cardiac remodelling. Interestingly, similar to clusterin and TnI, miR21 increased also immediately after infarction. miRNAs are key regulators of cardiovascular diseases and a variety of plasma miRNAs have been identified as stable circulatory biomarkers [45,46,47]. miR21 has previously been reported to be elevated in AMI patients and significantly correlate with cTnI and CK-MB [48]. Even though miRNAs are highly stable and rapidly released from damaged cells, the majority of miRNAs is neither disease nor organ specific. However, using a miRNA panel or combining (individual) miRNAs with well-known biomarkers such as hs-cTn might improve their diagnostic accuracy [49]. Interestingly, no correlation could be found between any of the early ischemia- or late remodelling-related biomarkers with clusterin.

In contrast with the other circulating factors (except TnI and miR21), clusterin proved to be a good marker for acute ischemia already 120 min after ischemia onset. This hypothesis is supported by the correlation of post AMI clusterin with LVEF and LVESV assessed by contrast ventriculography 30 min after reperfusion onset. Nevertheless, the mechanism of clusterin release can only be speculated. Clusterin is a heat shock protein-like intra- and extracellular chaperone and its expression is stimulated by cellular stress and tissue injury (e.g., ischemia, inflammation, apoptosis, oxidative stress, heat stress and ionising radiation) [7,9,50]. Extracellular clusterin stabilizes stressed proteins in a folding-competent state [8]. By clearing aggregating protein species and dead cells clusterin exerts anti-inflammatory and cytoprotective effects [9]. In the myocardium it protects the cardiomyocytes against apoptosis and promotes angiogenesis [51]. Our RNASeq data showed a significant upregulation of clusterin in the heart after 3 h and 3 days. Whereas gene expression compared to control decreased after 3 days in AMI tissue, we could observe an increase after 3 days in the remote region. Functional clustering of deregulated genes revealed an association of genes associated with apoptosis, inflammatory response to myocardial ischemia and angiogenesis. Immunohistochemical staining of human hearts after AMI showed increased expression of clusterin in the infarct zone at an early time point and increased expression in the peri-infarct zone in older infarct tissue, although not in healthy hearts [52]. Pavo et al. described increased clusterin expression in the infarct zone already five hours after the onset of myocardial ischemia and varying expression in the remote area, indicating a role in cardioprotection and restoring of cell function with a possible mediator role for intrinsic remote conditioning [51].

Clusterin expression is also associated with diabetes type II and high cholesterol levels, both being well known risk factors for atherosclerosis and AMI [8]. In our AMI model (in pigs not suffering from atherosclerosis, high cholesterol or diabetes) we could examine clusterin expression in acute myocardial ischemia without any confounding factors. Our results indicate that the increase in clusterin concentration immediately after AMI might be strongly associated to myocardial necrosis and not be caused by any underlying factors such as atherosclerosis and diabetes. However, a contributing part of those risk factors in humans cannot be excluded. This may also be the reason for sustained elevated clusterin levels in AMI patients that was previously reported [14]; however, this was not observed in our experiments with healthy animals. As already stated, hs-cTn levels are strongly influenced by a variety of cardiac and non-cardiac diseases that are common in patients at risk of AMI. Using hs-cTn as a single marker in patients with acute thoracic pain symptomatic as a tool to diagnose AMI might result in inconclusive results. We believe that plasma clusterin may serve as an additional biomarker in these patients further improving diagnostic accuracy of diagnostic standards.

Even though we provide evidence that plasma clusterin levels are regulated during controlled myocardial ischemia, several limitations to our study should be mentioned. Firstly, it is an observation study and no causality effects can be concluded, a mechanistic explanation of clusterin in AMI needs to be elaborated in further studies. Secondly, we measured clusterin levels in a small group of pigs; clinical applicability needs to be evaluated in a larger patient cohort. Third, we investigated clusterin dynamics only during a brief period after myocardial infarction; long term dynamics and a possible prognostic factor for mortality should be the subject of additional studies. Fourth, the current commercially available clusterin ELISA kits showed a relatively large scatter of data, which should be further refined.

## 4. Materials and Methods

### 4.1. Animals and Experimental Design

Domestic pigs (*n* = 39, weight 30–35 kg, female) underwent percutaneous coronary intervention (PCI) in order to induce catheter-based reperfused AMI. According to ESC guidelines [53,54] animals were premedicated with 250 mg aspirin and 300 mg clopidogrel and received daily doses of 100 mg aspirin and 75 mg clopidogrel during the follow up period. Functional assessment of the left ventricle and serial biomarker measurements were performed in 24 pigs, while myocardial gene expression of selected biomarker was performed 3 h and 3 days after AMI onset in five pigs of each time point.

Prior to left anterior descending artery (LAD) occlusion, the pigs received intramuscular injection of 12 mg/kg ketamine hydrochloride, 1 mg/kg xylazine and 0.04 mg/kg atropine as anaesthesia. Anaesthesia were deepened with isoflurane and O_2_ via mask and maintained with 1.5–2.5 vol% isoflurane, 1.6–1.8 vol% O_2_ and 0.5 vol% N_2_O via intratracheal tube. After induction of general anaesthesia, access to the right femoral artery was obtained through surgical preparation of the artery under sterile conditions and a 6-F introducer sheath (Medtronic, Minneapolis, MN, USA) was inserted. In total, 10,000 IU of heparin sodium were administered via the femoral sheath, and baseline haemodynamics were recorded. Selective angiography of the left coronary artery was performed by using a 6F guiding catheter (Medtronic, Minneapolis, MN, USA) with regular contrast media (Ultravist, Bayer, Leverkusen, Germany). After a baseline angiogram was analysed, a balloon catheter (2.75 m diameter, 8 mm length) (Abbot Vascular) was placed after the origin of the second diagonal branch. To induce AMI, the balloon was inflated with 5 atm for 90 min followed by deflation of the balloon resulting in reperfusion. Wounds were closed and anaesthesia was terminated by withdrawal of isoflurane. In this study, 1 g metamizole was applied intramuscularly (i.m.) as analgesia. Furthermore, 100 mg benzathine benzylpenicilline, 100 mg procaine benzylpenicillin and 200 mg dihydrostreptomycin-sulphate was given i.m. as antibiotic shielding. Heart rate, arterial blood pressure, electrocardiography, O_2_ saturation and temperature were monitored throughout the procedure.

The experiments were conducted at the Institute of Diagnostics and Oncoradiology, University of Kaposvar, Hungary. All animal facilities met the standards of the American Association for Accreditation of Laboratory Animal Care. Animal investigations were executed in accordance with the “Position of the American Heart Association on Research Animal Use” as adopted by the American Heart Association (AHA) on 11 November 1984. The study was approved by the Ethics Committee on Animal Experimentation at the University of Kaposvar, Hungary (EC: SOI/31/26-11/2014, approval date: 25 February 2014).

### 4.2. Blood Sampling

Peripheral blood samples were collected before occlusion (pre-AMI), and post-AMI (immediately before recovery from the anaesthesia, e.g., 120 min after start of coronary occlusion and 30 min after start of reperfusion), at 3 days and 3 weeks post AMI. Blood was centrifuged at 2000× *g* for 10 min and stored at −20 °C until further analyses were performed.

### 4.3. Measurement of Myocardial Necrosis and Functional Parameters by Contrast Ventriculography and Cardiac MRI

Biplane contrast ventriculography was performed 30 min after start of reperfusion, before the end of anaesthesia. Fifty mL contrast medium was infused by an injection pump at a rate of 12 mL/s via a 5F pig-tail catheter. LV volumina (LVEDV, LVSESV and LVEF) were calculated off-line by using the area-length methods (Quantcor LVA, Siemens, Germany). Magnification correction was calculated from the known internal diameter of the pig-tail catheter and the known distance between the mid chest of the animal and the radiography equipment, documented during the procedure. The end-diastolic and end-systolic contours were digitalized and traced automatically and LVEF was calculated.

At 3 days and 6 weeks after artificial myocardial infarction, cMRI + late enhancement (LE) was performed to assess myocardial necrosis, LVEF, LVEDV and LVESV. The cMRI + LE acquisition method and analyses have been described previously [55]. In accordance with the ethical principle of the 3 R (replace, reduce, refine) pre-AMI cMRI has not been performed as baseline left ventricular function parameters in pigs are similar to healthy humans and have been published previously [56,57].

### 4.4. Enzyme Linked Immunosorbent Assay (ELISA)

Plasma levels of clusterin (LS-F16326, LSBio, Seattle, WA, USA), neprilysin (MBS066263, San Diego, CA, USA), brain natriuretic protein (MBS706765, San Diego, CA, USA), neutrophil gelatinase-associated lipocalin (ab207924, Abcam, Cambridge, UK), osteopontin (LS-F23920, LSBio, Seattle, WA, USA), endothelin-1 (MBS2508397, San Diego, CA, USA) and troponin I, type 3 (SEA478Po, Cloud Clone, Houson, TY, USA) were assessed using commercially available ELISA Kits, which have been performed according to the manufacturers’ protocol. Absorbance was measured by Wallac Multilabel Counter 1420 (PerkinElmer, Waltham, MA, USA) at 450 nm. AutoOptical density values obtained at 450 nm (subtracted by plate background measured at 595 nm) were compared to the standard curve calculated from standards with a known concentration of the antigen. Measurements were performed in duplicates.

### 4.5. Transcriptomic Profiling

Detailed information is described in the Supplementary Material online and has been published previously [58]. Myocardial samples were obtained from the AMI and remote region, the latter was obtained from the opposite wall of the AMI region (mid lateral wall). Briefly, extracted total RNA of myocardial samples obtained on 3 h and 3 days after myocardial infarction onset were subjected to mRNA deep sequencing using the Illumina platform (San Diego, CA, USA). For mRNA fragmentation and enrichment NEB Next Poly(A) mRNA Magnetic Isolation Module (NEB, Ipswich, MA, USA) was used. Fragmented and primed mRNAs were reverse transcribed to cDNA. The NEBNext Ultra Directional RNA Library Kit (NEB, Ipswich, MA, USA) was used for cDNA library synthesising and enrichment. Finally, sequencing was performed on the HiSeq 2500 platform (mean depth: 15–20 million paired-end reads per sample) at the Core Facility Genomics (Medical University of Vienna, Vienna, Austria). Results were mapped to the pig transcriptome and analysed for statistically significant changes of individual genes. For analysis of biological relevance, groups were compared and significantly up- or downregulated genes were functionally clustered.

### 4.6. PCR

Total RNA was isolated from plasma using the miRNeasy Serum/Plasma Kit (Qiagen, Hilden, Germany). The RNA quantity and quality were measured with a nanodrop machine (Witec AG, Sursee, Switzerland). MiRNA was reverse transcribed to cDNA (Qiagen, Hilden, Germany) and expression was quantified by rtPCR (Applied Biosystems 7500 Real-Time PCR System, Life Technologies, Carlsbad, CA, USA). The primers for the target sequences were designed using Primer3 software version 4.1.0 (http://primer3.wi.mit.edu/primer3web_help.htm; Microsynth, Balgach, Switzerland). The relative gene expression level was calculated using the ΔCt method (i.e., expression level relative to an endogenous control). The expression changes were calculated relative to median expression at baseline.

### 4.7. Statistics

Data obtained were evaluated statistically using GraphPad Prism 6 software (GraphPad Software Inc., LA Jolla, CA, USA) and IBM SPSS Statistics version 23 (SPSS Inc., Chicago, IL, USA). Mixed linear models were used to compare parametric variables and for non-parametric variables after logistic transformation. Parametric variables were expressed as mean ± standard deviation (SD) and compared Student’s paired t-test. A Wilcoxon test and Friedman test were used to compare non-parametric, paired variables and expressed as median and interquartile range (IQR). Bonferroni correction was applied for multiple testing. For correlation of non-parametric variables Spearman’s rank correlation was used. All tests were performed in a two-sided manner. *p*-values equal or below 0.05 were considered statistically significant.

## 5. Conclusions

In conclusion, we have shown that plasma clusterin levels are associated with AMI in the early phase. In contrast to previous work, we did not observe sustained elevation of clusterin; however, this may be due to the fact that we could examine clusterin dynamics after AMI isolated from any concomitant diseases that are well known to be associated with altered clusterin expression.

## Supplementary Materials

Supplementary materials can be found at https://www.mdpi.com/1422-0067/21/13/4591/s1.

## Abbreviations

AMI	acute myocardial infarction
cMRI	cardiac magnetic resonance imaging
LE	late enhancement
LVEF	left ventricular ejection fraction
LVEDV	left ventricular end diastolic volume
LVESV	left ventricular end systolic volume
ELISA	enzyme linked immunosorbent assay
hs-cTn	high-sensitivity cardiac troponin
TnI	Troponin I
qPCR	quantitative polymerase chain reaction
ET-1	endothelin-1
LV	left ventricular
IQR	interquartile range
SD	standard deviation
LAD	left anterior descending artery
