# Supplementary material for: Early Elevation of Systemic Plasma Clusterin after Reperfused Acute Myocardial Infarction in a Preclinical Porcine Model of Ischemic Heart Disease
Source: Int J Mol Sci. 2020 Jun 28;21(13):4591. doi: 10.3390/ijms21134591 (PMC7369988; doi:10.3390/ijms21134591)
Supplement: Supplementary file 1 [file ijms-21-04591-s001.pdf]

Early elevation of systemic plasma clusterin after reperfused acute myocardial infarction in a preclinical porcine model of ischemic heart disease

Supplementary Information

Denise Traxler, Andreas Spannbauer, Patrick Einzinger, Julia Mester-Tonzcar, Dominika Lukovic, Katrin Zlabinger, Alfred Gugerell, LJubica Mandic, Mariann Gyöngyösi, Noemi Pavo

Supplementary Table 1. Cardiac muscle function and apoptosis related up- or downregulated genes in the AMI and remote region compared to controls.
Empty fields: non-significant expression change.

		AMI region		Remote region	
		3 hours	3 days	3 hours	3 days
		Log2 fold change			
Gene symbol	*Full Gene name*				
**Cardiac muscle function**					
DSC2	desmocollin 2	−0,851	−1,211	−0,897	
CACNA2D1	calcium voltage−gated channel auxiliary subunit alpha2delta 1	1,436	1,372	2.000	
KCNE1	potassium voltage-gated channel subfamily E regulatory subunit 1	1,112	1,357	0,770	
GJA5	Gap junction protein alpha 5	−2,419	−2,875	−2,534	−1,911
DSG2	desmoglein 2	−0,913	−0,989		−3,058
DSP	Desmoplakin	1,087	0,979	1,194	
JUP	Junction plakoglobin	−0,985	−0,500	−0,945	−0,808
CAV1	Caveolin 1	−0,880	−0,773	−0,904	−0,454
CACNA1G	Calcium voltage-gated channel subunit alpha1 G	−1,598		−0,904	
CSRP3	Cysteine and glycine rich protein 3	−1,324	−1,375	−1,253	−1,508
MYL4	Myosin light chain 4	−11,377	−9,982	−11,398	−9,741
MTOR	Mechanistic target of rapamycin kinase	0,912	0,831	1,116	0,480
MLC-2V	Myosin light chain 2V	7,595	7,382	7,677	6,675
TNNC1	Troponin C1, slow skeletal and cardiac type	0,567		0,674	
TNNI1	Troponin I1, slow skeletal type	−8,178	−7,028	−4,457	−6,122
MYL3	Myosin light chain 3	0,789		0,898	−0,782
SRF	Serum response factor	0,570			−0,774
ACTC1	Actin alpha cardiac muscle 1	-1,804	−2,424	−1,479	−1,588
SOD1	Superoxide dismutase 1		−0,463	−0,694	−0,636
CASQ2	Calsequestrin 2				−1,204
CAMK2D	Calcium/calmodulin dependent protein kinase II delta				−0,958
TCA	Titin-cap				−1,601
SRSF1	Serine and arginine rich splicing factor 1				0,826
ATP1B1	ATPase Na+/K+ transporting subunit beta 1				−0,454
FHOD3	Formin homology 2 domain containing 3				−0,937
MYPN	Myopalladin				−1,383
CFL2	Cofilin 2				−0,435
PRKAR1A	Protein kinase cAMP-dependent type I regulatory subunit alpha				0,636
LDB3	LIM domain binding 3				−1,332
ITGB1	Integrin subunit beta 1				1,750
MEF2C	Myocyte enhancer factor 2C	0,402	0,550		0,622
EZH2	Enhancer of zeste 2 polycomb repressive complex 2 subunit				2,362
GATA6	GATA binding protein 6				−0,981
INPP5F	Inositol polyphosphate-5-phosphatase F		1,259	0,945	1,764
HEY2	Hes related family bHLH transcription factor with YRPW motif 2	4,440	3,565	4,395	1,763
HRC	Histidine rich calcium binding protein				−1,312
ANK2	Ankyrin 2				−0,988
RYR2	Ryanodine receptor 2				−1,088
**Apoptosis**					
ADAMTSL4	ADAMTS like 4	−1,152		−0,913	−1,333
WT1	Wilms tumor homolog	3,360	4,032	4,039	3,496
REST	RE1−silencing transcription factor-like	−1,499	−1,429	−2,209	−1,206
MTCH1	Mitochondrial carrier 1	−0,647	−0,463	−0,757	
CTNNB1	Catenin beta 1	−0,393			0,497
MTCH2	Mitochondrial carrier 2	−0,408		−0,445	−0,530
PPID	Peptidylprolyl isomerase D	0,509			

APBB1	Amyloid beta precursor protein binding family B member 1	−1,233	−1,207	−1,135	−1,565
BNIP3L	BCL2 interacting protein 3 like	−0,653		−0,679	0,477
BMP4	Bone morphogenetic protein 4	−3,193	−2,979	−2,666	−2,703
FRZB	Frizzled related protein	−3,130		−4,099	−2,637
TGM2	Transglutaminase 2	−1,495	−0,920	−1,136	−0,769
SUDS3	SDS3 homolog, SIN3A corepressor complex componen	0,379	0,513	0,333	0,326
NCOA1	Nuclear receptor coactivator 1	0,403	0,419	0,524	
ING2	Inhibitor of growth family member 2	0,352			
APC	APC regulator of WNT signaling pathway	1,470	1,520	2,096	1,264
ING3	Inhibitor of growth family member 3	0,323		0,496	
CLU	Clusterin	1,899	1,285	1,574	2,263
SUPV3L1	Suv3 like RNA helicase	0,465		0,543	−0,549
CITED2	Cbp/p300 interacting transactivator with Glu/Asp rich carboxy−terminal domain 2	−0,783			−1,846
MIEN1	Migration and invasion enhancer 1	−0,443		−0,692	−0,583
SMO	Smoothened, frizzled class receptor	−0,785	−0,814	−1,084	−0,776
AHI1	Abelson helper integration site 1	−1,377			−1,235
TMBIM6	Transmembrane BAX inhibitor motif containing 6	−0,650		−0,769	0,693
TMF1	TATA element modulatory factor 1	0,460	0,591	0,907	0,847
FKBP8	FKBP prolyl isomerase 8	−0,448	−0,416	−0,368	
ATF5	Activating transcription factor 5	0,681		0,656	0,863
CSF1R	Colony stimulating factor 1 receptor	−2,725	−2,081	−1,957	
STAT5B	Signal transducer and activator of transcription 5B	−0,816	−0,812	−0,937	−1,142
MDM4	MDM4 regulator of p53	0,822	1,024	1,167	
NAA15	N−alpha-acetyltransferase 15, NatA auxiliary subunit	0,612	0,481	0,682	0,682
PIDD	P53-induced death domain protein 1	−1,223			1,215
ADAMTS20	ADAM metallopeptidase with thrombospondin type 1 motif 20	−2,863	−5,136		−5,961
HSPD1	Heat shock protein family D (Hsp60) member 1	0,471			0,723
BNIP3	BCL2 interacting protein 3	−0,689		−0,665	−1,295
SYCP2	Synaptonemal complex protein 2	1,096	0,939	1,313	1,914
GSK3B	Glycogen synthase kinase 3 beta	1,143	1,217	1,389	1,273
ZFAND6	Zinc finger AN1-type containing 6	−0,583	−0,440	−0,539	
PALB2	Partner and localizer of BRCA2	0,722		0,468	0,524
CYR61	Cellular communication network factor 1	1,327		2,020	1,207
PTEN	Phosphatase and tensin homolog	0,800	0,960	0,875	1,102
CBL	Cbl proto-oncogene	1,983	2,832	3,480	2,586
PLK3	Polo like kinase 3	1,479		1,348	3,125
MED1	Mediator complex subunit 1	1,026	1,163	1,347	0,540
NAA16	N-alpha-acetyltransferase 16, NatA auxiliary subunit	0,551		0,839	
MTDH	Metadherin	0,662	0,566	0,564	1,092
PLK2	Polo like kinase 2	1,061			
ATG5	Autophagy related 5	−1,093	−0,686	−1,145	−0,674
SON	SON DNA binding protein	0,772	0,872	0,894	1,577
IER3	Immediate early response 3	1,230			−1,508
AIFM2	Apoptosis inducing factor mitochondria associated 2		−0,611		
RARG	Retinoic acid receptor gamma		−1,036	−1,016	
MLLT11	MLLT11 transcription factor 7 cofactor		−1,480		−0,767
ZNF346	Zinc finger protein 346		−0,649	−0,598	−0,610
UBD	Ubiquitin D		1,160		
SMAD3	SMAD family member 3	0,603	0,568		
TNFRSF12A	TNF receptor superfamily member 12A	2,424	1,576	1,563	2,773
G0S2	G0/G1 switch 2	−3,514	−1,902	−2,051	−1,327
SGPP1	Sphingosine−1-phosphate phosphatase 1	−2,329	−2,494	−2,619	−1,676
IL33	Interleukin 33	−0,983	−1,571	−1,962	
TLR3	Toll like receptor 3		1,121		1,701
RBM5	RNA binding motif protein 5			−0,566	
PTGIS	Prostaglandin I2 synthase	−0,854	−0,885	−0,936	
APAF1	Apoptotic peptidase activating factor 1	−2,859	−1,560	−2,314	−1,042
TFPT	TCF3 fusion partner	0,848		0,681	

SENP1	SUMO specific peptidase 1	0,452	1,050	0,644	1,275
DAPL1	Death associated protein like 1	7,686	7,214	7,187	3,916
ANXA6	Annexin A6	−0,611	−0,477	−0,527	
SHQ1	SHQ1, H/ACA ribonucleoprotein assembly factor				0,589
APBB2	Amyloid beta precursor protein binding family B member 2				0,552
SAV1	Salvador family WW domain containing protein 1				−0,406
ENDOG	Endonuclease G				−1,491
IRF5	Interferon regulatory factor 5				2,920
ANKRD1	Ankyrin repeat domain 1				0,875
RARB	Retinoic acid receptor beta				−0,687
ITGA6	Integrin subunit alpha 6				0,787
DCUN1D3	Defective in cullin neddylation 1 domain containing 3				0,668
TOP2A	DNA topoisomerase II alpha				3,470
UTP11L	UTP11 small subunit processome component				0,705
DNAJA1	DnaJ heat shock protein family (Hsp40) member A1				0,839
BIN1	Bridging integrator 1				0,657
NEURL	Neuralized E3 ubiquitin protein ligase 1				1,852
BAX	BCL2 associated X, apoptosis regulator				1,512
PRKDC	Protein kinase, DNA−activated, catalytic subunit				0,466
KIAA1967	cell cycle and apoptosis regulator 2				0,368
COPS5	COP9 signalosome subunit 5				−0,382
NET1	Neuroepithelial cell transforming 1				1,433
RPS6KA2	Ribosomal protein S6 kinase A2				1,570
HSPCB	Heat shock protein 90 alpha family class B member 1				1,324
PRNP	Prion protein				−0,747
AATF	Apoptosis antagonizing transcription factor				0,706
MYC	MYC proto−oncogene, bHLH transcription factor				2,336
PTK2B	Protein tyrosine kinase 2 beta				1,714
GLI2	GLI family zinc finger 2				0,672
STAT3	Signal transducer and activator of transcription 3				1,254
DNAJC3	DnaJ heat shock protein family (Hsp40) member C3				1,025
HSP90B1	Heat shock protein 90 beta family member 1				1,689
RPS3A	Ribosomal protein S3A				0,968
CD38	CD38 molecule				0,759
AREL1	Apoptosis resistant E3 ubiquitin protein ligase 1				0,471
AURKA	Aurora kinase A				1,968
SPHK1	Sphingosine kinase 1				2,348
SERPINB2	Serpin family B member 2				1,696
CIB1	Calcium and integrin binding 1				1,094
CAT	Catalase				−0,869
THOC6	THO complex 6				1,666
FAIM	Fas apoptotic inhibitory molecule				−0,965
DAD1	Defender against cell death 1				0,572
GRK5	G protein−coupled receptor kinase 5				−0,793
SIN3A	SIN3 transcription regulator family member A				0,443
PDCD10	Programmed cell death 10				0,911
LIMS2	LIM zinc finger domain containing 2				−0,984
DAB2	DAB adaptor protein 2			0,576	1,858
DDB1	Damage specific DNA binding protein 1				0,513
CDC2	Cyclin dependent kinase 1				3,185
CLN8	CLN8 transmembrane ER and ERGIC protein				0,874
MDM2	MDM2 proto-oncogene				0,515
ANGPT4	Angiopoietin 4				2,426
PIM1	Pim-1 proto-oncogene, serine/threonine kinase				1,412
CTSH	Cathepsin H				3,271
PLAC8	Placenta associated 8		2,828		2,163
GLO1	Glyoxalase I				−0,420
MRE11A	MRE11 homolog, double strand break repair nuclease				0,394
RARA	Retinoic acid receptor alpha				0,585
HSPA5	Heat shock protein family A (Hsp70) member 5				1,688
CTH	Cystathionine gamma−lyase		1,833	1,954	1,861
BCL3	BCL3 transcription coactivator				1,364

ASNS	Asparagine synthetase (glutamine-hydrolyzing)				1,248
TP53	Tumor protein p53				1,068
IGFI	Insulin like growth factor 1				1,206
TP53BP2	Tumor protein p53 binding protein 2	−0,764			−0,661
PPM1F	Protein phosphatase, Mg2+/Mn2+ dependent 1F				0,412
PPP1R13B	Protein phosphatase 1 regulatory subunit 13B				−1,582
DDX3X	DEAD−box helicase 3 X-linked				1,246
BCAP31	B cell receptor associated protein 31				0,636
PDCD5	Programmed cell death 5				−0,491
HMGB1	High mobility group box 1				0,901
TNFSF10	TNF superfamily member 10			−1,211	-0,864
RHOA	Ras homolog family member A				1,219
CYCS	Cytochrome c, somatic		-0,547		−1,164
BID	BH3 interacting domain death agonist				0,889
ITM2C	Integral membrane protein 2C	-0,814		-1,206	0,561
GRP-58	Protein disulfide-isomerase				1,930
TRAF2	TNF receptor associated factor 2				0,727
HYAL2	Hyaluronidase 2				0,868
RIPK1	Receptor interacting serine/threonine kinase 1				1,577
LTBR	Lymphotoxin beta receptor				0,779
BCLAF1	BCL2 associated transcription factor 1	0,504		0,724	0,778
SEPTIN4	Septin 4				−0,632
RIPK3	Receptor interacting serine/threonine kinase 3				1,462
SLC9A3R1	SLC9A3 regulator 1				1,496
LMNA	Lamin A/C				1,277
RPS6KB1	Ribosomal protein S6 kinase B1	0,263			0,288
ZYMND11	Zinc finger MYND-type containing 11				−0,254
SRC	SRC proto-oncogene, non-receptor tyrosine kinase				0,415
GCLC	Glutamate-cysteine ligase catalytic subunit				1,669
ITGAV	Integrin subunit alpha V				0,658
LGALS3	Galectin 3				4,387
HTT	Huntingtin				1,456
PSME3	Proteasome activator subunit 3	0,313			0,484
ACVR1	Activin A receptor type 1	-0,463	-0,753	-0,812	−1,055
NRP1	Neuropilin 1				0,456
FIGNL1	Fidgetin like 1				1,232
MAPK8IP1	Mitogen−activated protein kinase 8 interacting protein 1				1,223
HELLS	Helicase, lymphoid-specific				2,325
IVNS1ABP	Influenza virus NS1A binding protein	−0,900	−0,784		−1,771
VDAC2	Voltage dependent anion channel 2	−0,600	−0,615	−0,654	−1,170
TNFRSF1B	TNF receptor superfamily member 1B				2,221
CASP9	Caspase 9				1,322
NFATC4	Nuclear factor of activated T cells 4				1,089
PIGBRCA1	BRCA1 DNA repair associated				1,478
TNFRSF1A	TNF receptor superfamily member 1A				1,287
ERCC6	ERCC excision repair 6, chromatin remodeling factor	0,796	1,315	1,360	0,920
MLH1	MutL homolog 1			−0,529	−0,603
POLB	DNA polymerase beta				−0,429
CREB3	CAMP responsive element binding protein 3				0,579
LRRK2	Leucine rich repeat kinase 2				1,442
SYVN1	Synoviolin 1	−0,618			0,430
PTP1B	Protein tyrosine phosphatase non−receptor type 1		1,164	0,753	0,955
PARK7	Parkinsonism associated deglycase				−0,464
RGL2	Ral guanine nucleotide dissociation stimulator like 2				0,262

Supplementary Materials and Methods

Gene expression profiling by NGS

Tissue samples from the AMI region and remote region 3 hours and 3 days after acute myocardial infarction onset of all surviving animals were stored in RNAlater (Qiagen, Germany) at −80°C. Total RNA was isolated by using the RNeasy Microarray Tissue Mini Kit (Qiagen, Germany). RNA quality was checked on RNA Nano chips using the Agilent 2100 Bioanalyzer platform (Agilent Technologies).

For RNA-sequencing, strand specific libraries for paired end sequencing on a HiSeq 2500 (Illumina, San Diego, CA, USA) were prepared from poly-A enriched RNA (NEBNext Poly(A) mRNA Magnetic Isolation Module, NEB, Ipswich, MA, USA) starting with 500 ng total RNA and using the NEBNext Ultra Directional RNA Library Prep Kit for Illumina (NEB). Libraries were quality controlled on a Fragment Analyzer (Advanced Analytical Technologies, Ames, IA, USA) and quantified by digital droplet PCR (QX100™ Droplet Digital™ PCR System, Bio-Rad, Hercules, CA, USA) and the ddPCR Library Quantification Kit for Illumina (Bio-Rad). Eight to ten libraries were pooled equimolarly for one HiSeq 2500 lane and each library was sequenced 50 base pairs paired end to a mean depth of 19.9 million (SD 6.1 million) paired reads. After demultiplexing, raw reads were quality controlled by FastQC (http://www.bioinformatics.babraham.ac.uk/projects/fastqc/) and sub sequentially mapped to the *Sus scrofa* genome (Sscrofa10.2) by the RNA-Seq Unified Mapper (RUM v2.0.4) [1]. HTSeq software was used to count reads into Sus scrofa gene model. [2]. Following analyses were done in R v4.0.0 (R Core Team, R Foundation for Statistical Computing) using R-package dnet v1.1.7 [3] and biomaRt v2.44.0 [4].

I.E. raw read counts were log2 counts per million (counts + 0.5) / (lib. size + 1) 1e+06) transformed and gene weights were calculated for use in subsequent statistical testing. EdgeR package was utilised to identify differentially expressed genes (DEGs) and read count values were normalised and trimmed to remove genes with a low expression. Statistically significantly deregulated genes were determined by linear models fitted to the corresponding contrasts (e.g. AMI versus control in each heart region). Animals and regions were used as confounding factors in the models. The estimated coefficients and standard errors for all contrasts were used for calculation of moderated t-statistics, moderated F-statistics, and the log-odds of differential expression by empirical Bayes shrinkage of the standard errors towards a common value. alse discovery rate (FDR) below 5% was accounted as statistically significant. FDR cut-off of 10% was considered for some contrasts, for relevant gene lists for biomedical interpretation. Deregulation of the single gene was represented by the log2 fold changes. Family-wise error rate (FWER) correction and false discovery rate (FDR) correction were applied to determine the regulation of genes and pathway topology with a cut- off of 5 %.

For defining the high scoring STRING db v9.1 [5] sub-networks for all analyzed contrasts the *Sus scrofa* Ensembl gene identifiers were mapped to the HUGO gene symbols (HUGO Gene Nomenclature Committee (HGNC), http://www.genenames.org/) using R-package biomaRt. To determine the high scoring sub-networks for each contrast the mappable (*Sus scrofa* Ensembl IDs -> HUGO symbols) with their corresponding raw p-values were projected on the STRING network of known and predicted protein interactions and the high scoring sub-network determined. The heuristic algorithm is supposed to find the maximum scoring sub-network from an input network and scores (raw p-values corresponding to the desired contrast) imposed on its nodes. The input scores imposed on the nodes in the input graph can be divided into two parts: the positive nodes and the negative nodes by using a tunable cut-off. The searching for the maximum scoring sub-network is deduced to find the connected sub-network maximizing the positive nodes but minimizing the negative nodes. To this end, a heuristic search is used with minimizes the sum of p-values and maximizes the number of edges in the network. In our case p-value cut-offs were chosen to get high-scoring sub-networks of at least 50 nodes. Finally, nodes (representing genes with their HUGO gene symbols) in the high-scoring sub-

networks were colored with the corresponding log2 fold changes between both conditions interrogated in the corresponding contrast.

Biological interpretation of significant gene lists was performed using the Database for Annotation, Visualization and Integrated Discovery (DAVID) v6.8 [6].

Supplementary References

1. Grant, G.R.; Farkas, M.H.; Pizarro, A.D.; Lahens, N.F.; Schug, J.; Brunk, B.P.; Stoeckert, C.J.; Hogenesch, J.B.; Pierce, E.A. Comparative analysis of RNA-Seq alignment algorithms and the RNA-Seq unified mapper (RUM). *Bioinformatics* **2011**, *27*, 2518–2528, doi:10.1093/bioinformatics/btr427.
2. Anders, S.; Pyl, P.T.; Huber, W. HTSeq—a Python framework to work with high-throughput sequencing data. *Bioinformatics* **2015**, *31*, 166–169, doi:10.1093/bioinformatics/btu638.
3. Fang, H.; Gough, J. The `dnet' approach promotes emerging research on cancer patient survival. *Genome Medicine* **2014**, *6*, 64, doi:10.1186/s13073-014-0064-8.
4. Durinck, S.; Spellman, P.T.; Birney, E.; Huber, W. Mapping identifiers for the integration of genomic datasets with the R/Bioconductor package biomaRt. *Nat. Prot.* **2009**, *4*, 1184–1191, doi:http://www.nature.com/nprot/journal/v4/n8/suppinfo/nprot.2009.97_S1.html.
5. Franceschini, A.; Szklarczyk, D.; Frankild, S.; Kuhn, M.; Simonovic, M.; Roth, A.; Lin, J.; Minguez, P.; Bork, P.; von Mering, C., et al. STRING v9.1: protein-protein interaction networks, with increased coverage and integration. *Nucleic Acids Res.* **2013**, *41*, D808–D815, doi:10.1093/nar/gks1094.
6. Jiao, X.; Sherman, B.T.; Huang, D.W.; Stephens, R.; Baseler, M.W.; Lane, H.C.; Lempicki, R.A. DAVID-WS: a stateful web service to facilitate gene/protein list analysis. *Bioinformatics* **2012**, *28*, 1805–1806, doi:10.1093/bioinformatics/bts251.
